# Increasing collaborative research output between early-career health researchers in Africa: lessons from the CARTA fellowship program

**DOI:** 10.1080/16549716.2020.1768795

**Published:** 2020-06-08

**Authors:** Dieudonne Uwizeye, Florah Karimi, Emmanuel Otukpa, Moses W. Ngware, Hesborn Wao, Jude Ofuzinim Igumbor, Sharon Fonn

**Affiliations:** a School of Governance, University of Rwanda, Kigali, Rwanda; b African Population and Health Research Center, Nairobi, Kenya; c School of Public Health, University of Witwatersrand, Johannesburg, South Africa

**Keywords:** Collaborative publications, networks, doctoral intervention, post-doctoral intervention, Africa

## Abstract

In 2008 nine African Universities and four African research institutions, in partnership with non-African institutions started the Consortium for Advanced Research Training in Africa (CARTA) to strengthen doctoral training and research capacity on health in Africa. This study describes particular aspects of the CARTA program that promotes collaboration between the PhD fellows in the program, and determines the patterns of collaborative publications that resulted from the intervention. We reviewed program monitoring and evaluation documents and conducted a bibliometric analysis of 806 peer-reviewed publications by CARTA fellows published between 2011 and 2018. Results indicate that recruiting multidisciplinary fellows from various institutions, encouraging registration of doctoral-level fellows outside home institutions, and organizing joint research seminars stimulated collaborative research on health-related topics. Fellows collaborated among themselves and with non-CARTA researchers. Fellows co-authored 75 papers (10%) between themselves, of which 53 (71%) and 42 (56%) included fellows of different cohorts and different disciplines respectively, and 19 (25%) involved fellows of different institutions. CARTA graduates continued to publish with each other after graduating – 11% of the collaborative publications occurred post-graduation – indicating that the collaborative approach was maintained after exiting from the program. However, not all fellows contributed to publishing collaborative papers. The study recommends concerted effort towards enhancing collaborative publications among the CARTA fellows, both doctoral and post-doctoral, which can include holding research exchange forums and collaborative grant-writing workshops.

## Background

Africa requires well trained and networked researchers, capable of responding to challenges in public and population health [1–3]. Interdisciplinary collaborative research is particularly essential for public and population health to address its complexity, to deal with the multifaceted determinants of health and to describe and understand the local context in which interventions are proposed or evaluated [4,5]. Interdisciplinary collaborative research potentially leads to high-quality scholarly productivity [3,6,7] as it usually brings together people with various skills and experiences to research and potentially enriches the quality of research [8]. Researchers in health sciences generally conduct collaborative research and publish with multiple authors compared to humanities and social sciences [9]. However, the collaboration between health researchers from different countries, institutions, and disciplines in Africa remains low [4,10,11]. Therefore, training programs that create opportunities for collaboration can make a valuable contribution to the African health research landscape [3,12,13].

An intervention that is promoting collaborative health research in Africa is the Consortium for Advanced Research Training in Africa (CARTA), established in 2008. CARTA currently consists of eight African universities, four African research institutions, and eight non-African academic and research institutions. The consortium is funded by multiple institutions interested in higher education and research on health in Africa. It is an African initiated and led south-south-north consortium committed to, among other things, training high-quality doctoral-level scholars which emphasises cross-disciplinary, cross-institutional, and cross-cohort collaboration. We define CARTA as south-south-north consortium because it was initiated between African partners who then invited interested non-African partners to join the consortium [2] to create a partnership that encourages collective and reciprocal learning [8]. The consortium membership is presented in Table 1.

**Table 1.** CARTA partner institutions.

CARTA members		
*African Universities*	*African Research Centers*	*Non-African Partners*
Makerere University, Uganda Moi University, Kenya Obafemi Awolowo University, Nigeria University of Ibadan, Nigeria University of Malawi, Malawi University of Nairobi, Kenya University of Rwanda, Rwanda University of the Witwatersrand, South Africa	African Population & Health Research Center (APHRC), Kenya Agincourt Population and Health Unit, South Africa Ifakara Health Institute (IHI), Tanzania KEMRI/Wellcome Trust Research Program, Kenya	Canadian Coalition for Global Health Research, Canada Swiss Tropical and Public Health Institute, Switzerland University of Gothenburg, Sweden Umeå University, Sweden University of Warwick, UK Brown University, Providence, USA ESE:O, Santiago, Chile

CARTA competitively recruits a maximum of four individuals from early-career university academics, and one from a research centre of each African partner institutions for PhD fellowships on an annual basis. The consortium has a deliberate approach to building a network between the PhD fellows in the design of the program. The CARTA approach is described in detail in earlier publications [2,14]. Here we describe critical aspects of the programme developed to promote collaboration between PhD fellows thereafter we assess if the CARTA program has stimulated cross-institutional, cross-cohort, and cross-disciplinary research output between CARTA fellows by assessing their publication output.

## Design of the CARTA program

CARTA recruits PhD fellows from any discipline as long as their proposed research topics address public and population health. CARTA brings the fellows together regularly through four four-week Joint Advanced Seminars (JAS) which supplement the discipline-specific PhD training they get at the universities where they register for their PhDs. The JAS curriculum is multidisciplinary, and it is jointly developed by the senior academics and researchers from both the African and non-African consortium members.

The JAS expose the fellows to the contribution of collaborative research across disciplines and geographies to understanding and solving health issues; engages fellows in teamwork across disciplines and institutions; encourages collaborative approaches to problem-solving; and creates networks between fellows from different partner institutions, and between fellows from different disciplines within and between the partner institutions. Fellows in the first year of their doctoral fellowship are given opportunities to interact and collaborate with those in the final year of their doctoral fellowships as the first and fourth JAS are held concurrently and at the same venue with purposeful curriculum overlap. The more senior group, who are attending their last JAS, interact with and peer-mentor those attending their first JAS. This is described in more detail elsewhere [2,14,15]. JAS sessions create formal and informal opportunities for fellows to cultivate interpersonal relationships. In the course of the fellowship, fellows have communicated with each other spontaneously by setting up cohort-created forums such as emails and WhatsApp groups. JAS two and three have been held at the University of the Witwatersrand and Ibadan respectively. JAS one and four, which are concurrent, have been held in East Africa and have moved between Kenya, Tanzania and Uganda.

CARTA also encourages fellows to register for their doctoral degree programs in any of the African consortium partner universities and facilitates this by paying their tuition fees and providing a more substantial stipend to make being away from home affordable. The primary aim of this cross-registration is to allow fellows to experience an academic environment other than their own, and to develop new networks. By 2018, fifty-two fellows (28%) had registered for their doctoral degrees at institutions other than their own [16]. Some fellows thus have a dual affiliation, their institution of employment (home institution) and the institution where they register for their degree (host institution).

## Postdoctoral interventions

The CARTA postdoctoral interventions are in the form of post-doctoral awards, which include postdoctoral fellowships, research re-entry grants, and graduate workshops. The interventions strengthen research collaboration among the fellows, institutions, and between CARTA fellows and non-CARTA researchers. Through postdoctoral awards, CARTA fellows receive support to work within a research team with experienced mentors to facilitate entering into a research network in their field of speciality after completion of their doctoral training [17]. By 2018, sixty-two of the fellows had graduated, and CARTA had awarded eighteen postdoctoral awards from cohorts 1, 2, and 3, the cohorts which have most of the graduates.

Further, CARTA organizes graduate workshops to support fellows to develop research proposals. These workshops are designed as an individual exercise rather than joint grant writing. Nonetheless, it provides a forum for CARTA graduates to interact and has the potential for further networking and research collaboration between fellows.

## Methodology

### Study design

This study used document analysis and bibliometric review of the CARTA fellows’ peer-reviewed publications from 2011 to 2018. The study considered cohorts one to eight, recruited from 2010 to 2017. The ninth and tenth cohorts of CARTA fellows, recruited in 2018 and 2019 respectively, were excluded from the study as they were unlikely to have published within the CARTA doctoral intervention period.

### Data collection and management

**Figure 1.**
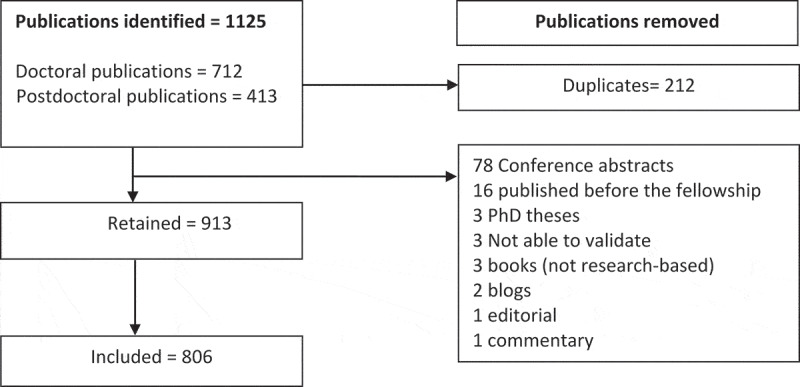
Selection of publications analysed from the database.

All fellows complete biannual reports as well as other surveys as part of CARTA’s routine monitoring and evaluation system. Data from this source was used to collate demographic information about the fellows. The demographic information was used to link the publication titles to the authors and their characteristics, including their cohort, institutional affiliation, and their disciplinary background. Figure 1 shows the process followed to include and exclude publications that fellows reported to CARTA.

We reviewed the 1125 publications reported by CARTA fellows to the CARTA Monitoring and Evaluation office. We validated the accuracy of citation by checking each publication online through PubMed, Google Scholar, or ResearchGate databases. In cases where the publications were not available in these sources, we contacted the respective CARTA fellow to verify the full citation. We omitted from the study publications that were not peer-reviewed, and publications that could neither be accessed online nor verified as accurate citations by the respective authors. Further, we excluded books, theses, editorials, commentaries, and blog publications, as we could not establish whether the publication was a result of a peer-review process. We remained with 806 publications, as shown in Figure 1.

### Analysis

The unit of analysis is the publication, from which we collected authorship information and the year of publication. The CARTA fellow appearing first on the list of authors was used as the reference author for analysis. We aligned each publication with the cohort number, gender, and home and host institution of the reference author. We recorded all publications and noted where CARTA fellows are co-authors on publications with other people and where CARTA fellows published with each other which we call collaboration. We also tracked the year of completion for each fellow which refers to the year of thesis defence or the year a fellow received a letter of completion.

We recorded the authorship information to determine whether the CARTA fellows were working with each other by cohort, institution, and disciplines, or collaborating with non-CARTA researchers to publish. We also analysed gender differences in the patterns of co-authorship. We used the date of the submission of the manuscript to classify publications into two main scenarios: publications resulting from the doctoral intervention (doctoral publication), and publications resulting from the postdoctoral intervention (postdoctoral publication). Doctoral publications were defined as those submitted for publication within the CARTA doctoral fellowship period and up to one year after completion. The postdoctoral publications are those submitted after this period. Similarly, we classified the publications resulting from the postdoctoral intervention in two scenarios: manuscripts submitted while the CARTA fellow received a CARTA-funded postdoctoral fellowship or a re-entry research grant, and a manuscript submitted when the fellow had not received any of the CARTA postdoctoral fellowships.

To analyse institutional collaborations, we associated the publications submitted during the fellowship with the host institution, and with the home institution for those submitted after the fellowship.

#### Academic disciplinary fields

We used the International Standard Classification of Education (ISCE) [18] as a reference document to assign the academic disciplinary field of each fellow. Then, we referred to the description of the MD-PhD disciplines by O’Mara et al. [19], and the size of the available data to make the classification. For example, ISCE [18] classified ‘Health’ as the main field of science under which clinical medicine, medical services, nursing, and dental services are the subfield. In this paper, we present each of the subfields separately because most of the available data were in the health field. Following the discussion by O’Mara et al. [19], we subdivided ‘Medicine’, a subfield of ‘Health’ category, into clinical medicine, medical services and paramedical sciences. The last two, medical services and paramedical sciences, were made the same category as they were very close to medical fields. Also, we presented the subfield of ‘demography and population studies’, initially part of social sciences, and ‘public health’, initially part of medical services, each as independent subfields because we had a significant number of publications in that field. A degree of granularity was chosen to understand better if collaboration occurred. Table 2 illustrates the details of how we classified the fields of study of the CARTA fellows and the number of fellows in each of the disciplinary areas.

**Table 2.** Classification of the public and population health-related fields of study by CARTA fellows (2011–2018).

Classification	Details of the fields of study by CARTA fellows	Number of fellows per discipline
(i)*Public health*	Public health; health systems and policy; community health; health promotion; adolescent health; epidemiology and public health	46
(ii)*Medical service and paramedical sciences*	Human nutrition; exercise science; kinesiology and physiotherapy; medical physiology; immunity and infections; medical bacteriology; biomedical laboratory; pharmacy; pharmacognosy; dentistry; molecular genetics; audiology; ophthalmology; neuroscience; microbiology; occupational therapy	29
(iii)*Epidemiology and biostatistics*	Mathematics and statistics; epidemiology and biostatistics; disease epidemiology	24
(iv)*Demography and population studies*	Demography and population studies; demography and social statistics; population studies; social statistics	16
(v)*Life and environmental health sciences*	Environmental health; zoology; human geography; botany; one health; microbiology; chemistry; biochemistry.	15
(vi)*Social and behavioural sciences*	Medical anthropology; sociology; psychology; social development; clinical psychology.	15
(vii)*Nursing*	Obstetrics and reproductive health nursing; general nursing; maternal health nursing; midwifery; mental health nursing.	13
(viii)*Clinical medicine*	Pediatrics & child health; clinical medicine; community medicine; internal medicine; oncoplastic surgery	11
(ix)*Others*	Literature; linguistics; library sciences; information sciences; education; communication; political science and public administration; human resource management; strategic management; environmental planning and management; design; civil engineering.	16

Ultimately, eight major public and population health-related disciplinary areas were identified. These were demography and population studies; life and environmental health sciences; epidemiology and biostatistics; public health; nursing; social and behavioural sciences; clinical medicine; and medical service and paramedical sciences. The fields of study which could not be classified into any of these fields were classified as ‘other’ as presented in Table 2.

## Results

Table 3 shows the number of fellows per institution in each cohort and the academic disciplinary area of fellows from Cohort 1 to Cohort 8.

**Table 3.** Number of fellows per institution and the academic disciplinary area in each cohort.

	Total	Cohort 1	Cohort 2	Cohort 3	Cohort 4	Cohort 5	Cohort 6	Cohort 7	Cohort 8
Total number of fellows	185	20	18	22	27	22	24	26	26
**African partner institutions**									
University of Ibadan	**28**	2	3	4	3	4	4	4	4
University of Malawi	**26**	4	4	3	2	3	3	4	2
Obafemi Awolowo University	**24**	3	1	3	3	2	4	4	4
University of Witwatersrand	**23**	3	2	2	4	2	4	3	3
Makerere University	**19**	-	4	1	4	3	2	2	3
University of Nairobi	**18**	1	1	2	2	2	3	3	4
Moi University	**17**	2	1	4	4	3	-	1	2
University of Rwanda	**15**	3	-	1	1	1	2	3	4
University of Dar es Salaam	**5**	-	1	2	2	-	-	-	-
Ifakara Health Institute	**5**	1	-	-	1	1	1	1	-
APHRC	**5**	-	1	-	1	1	1	1	-
Agincourt Research Institute	**1**	1	-	-	-	-	-	-	-
**Academic Discipline**									
Public health	**46**	1	3	8	9	7	6	4	8
Medical service sciences	**29**	2	2	3	4	3	5	4	6
Epidemiology/Biostatistics	**24**	7	8	3	1	-	1	3	1
Demography and population studies	**16**	6	2	2	-	1	2	2	1
Social and behavioural sciences	**15**	-	-	2	6	-	2	5	-
Life and Environmental health sciences	**15**	2	2	3	-	2	2	1	3
Nursing	**13**	-	1	1	1	3	3	2	2
Clinical medicine	**11**	1	-	-	1	2	1	3	3
Other	**16**	1	-	-	5	4	2	2	2

Institutional representation in each cohort is not equitably distributed because recruitment is based on merit. Cohort 4 has the highest institutional representation with eleven different institutions represented, followed by Cohort 5 and Cohort 7, with ten institutions represented in each.

Overall, the highest number of CARTA fellows are drawn from ‘public health’ (itself often multidisciplinary in nature), which is followed by ‘medical service and paramedical sciences’. Further, Cohort 6 and Cohort 7 can be singled out as the cohorts with the broadest range of disciplines represented. The category described as ‘other,’ which included 16 fellows, illustrates the wide range of disciplines that are not traditionally linked to public and population health but were nonetheless attracted to CARTA. The disciplines were as diverse as literature and linguistics and political science to design and civil engineering.

## Patterns of collaboration in CARTA fellows’ publications

Table 4 presents the CARTA fellows’ collaborative publications with other researchers and with each other, by gender. The table presents the fellows’ publication output and specifically co-publication output between cohorts, disciplines and universities. As illustrated, 140 (76%) of the fellows published a total of 806 papers, and 777 of those publications (more than 96%) were multi-author papers involving 136 fellows, and 29 papers were single-authored produced by 18 fellows. Among the 777 multi-author papers, CARTA fellows were the first authors on 318 papers (41%).

**Table 4.** Publications by gender and the number of fellows who reported publication output.

Publications by CARTA fellows Overview		Total number of papers	Number of papers reported by		Number of fellows who reported the papersa		
			Female	Male	Total (n = 185)	Female (n = 102)	Male (n = 83)
*Total number of publication*		806	303	503	140	70	70
*Collaborative publications*		777 (96.4%)	288 (95%)	489 (97%)	136	68	68
*Single authored publications*		29 (3.6%)	15 (5%)	14 (3%)	18	8	10
**Collaborative papers with fellows and non-CARTA researchers**							
**Total number of publications**	**777**		**288**	**489**	**136**	**68**	**68**
***Publication leadership***							
*A fellow is the lead author*		318 (41%)	131 (45%)	187 (38%)	112	55	57
*A fellow is not the lead author*		459 (59%)	157 (55%)	302 (62%)	107	48	59
**Number of fellows in the publication team**							
*1*		702 (90%)	268 (93%)	434 (89%)	132	65	67
*2*		64 (8%)	18 (6%)	46 (9%)	32	12	20
*3*		10 (1.3%)	2 (1%)	8 (1.8%)	8	2	6
*20*		1 (.7%)	0 (.0%)	1 (.2%	1	0	1
***Period of publication***							
*During the PhD training period*		626 (81%)	246 (85%)	380 (78%)	134	67	67
*After the PhD training period, with no postdoc grant*		86 (11%)	36 (13%)	50 (10%)	15	7	8
*During/after postdoctoral research grant*		65 (8%)	6 (2%)	59 (12%)	11	3	8
**Patterns of cross-CARTA fellows collaborative publications**							
**Total number of publications** ***Same cohort***		***75***	***20***	***55***	***34***	***12***	***22***
*No*		*53*	*13*	*40*	*29*	*10*	*19*
*Yes*		*22*	*7*	*15*	*10*	*3*	*7*
***Same institution***							
*No*		*19*	*4*	*15*	*12*	*3*	*9*
*Yes*		*56*	*16*	*40*	*28*	*10*	*18*
***Same discipline***							
*No*		42	9	33	23	7	16
*Yes*		33	11	22	19	7	12
***Type of intervention received***							
*Doctoral intervention only*		*46*	*13*	*33*	*32*	*11*	*21*
*With a postdoc intervention*		*19*	*2*	*17*	*5*	*2*	*3*
*Graduated, with no postdoc intervention*		*10*	*5*	*5*	*6*	*2*	*4*

^a^The number of the type of publication per fellow was not mutually exclusive- multiple authors on a single publication could each have reported the output; consequently, the number of fellows does not add up to the total number of publications.

Further, 626 multi-author publications (81%) were produced during the doctoral intervention period, with only 8% having been produced during the postdoctoral intervention period. CARTA graduates who had not received the CARTA postdoctoral grant or re-entry grant continued to publish and produced 11% of the collaborative publications.

CARTA fellows collaborated with each other and produced 75 publications, which was approximately 10% of the collaborative papers. Among these 75 papers, fellows from different cohorts co-published 71% of these papers, and 25% of publications reflect cross-institutional collaboration. Among the 75 papers on which fellows were co-authors with other, 20 were reported by female fellows against 55 reported by male fellows.

The data further indicates that fellows collaborated across disciplines (56% of the papers); the rest reflect collaboration within the same disciplines. The majority (61%) of the between-CARTA-fellows collaborative papers were produced during the doctoral intervention period. We also identified one publication which involved all fellows from one cohort describing their experience of CARTA [15], and one cross-cohort and cross-disciplinary collaborative paper which included a CARTA senior academic, describing the engagement between librarians brought together by CARTA [20].

## Discussion

We described the interventions of the CARTA program that aimed to inspire fellows to work collaboratively. We used the joint publications between CARTA fellows as an output measure. Overall we found that writing multiple author papers is common among CARTA fellows and graduates as is to be expected given usual practice in the disciplines that usually contribute to health research. Also, we observed that CARTA fellows co-published beyond their traditional research fields in multidisciplinary, cross-cohort and cross-institutional teams [15,20]. We have shown that CARTA fellows are writing with each other, mainly within their institutions but across disciplines and CARTA cohorts. We have also illustrated that CARTA fellows from different institutions are publishing with each other, but this is less common.

However, we observed that nearly a quarter of the fellows had not published in the period under review. Most of the CARTA fellows are academics employed in universities, and a minority are employed in African research institutions. It is reasonable, therefore, to expect that some of them are research active, in particular those employed in research institutions. This would explain the high number of publications with other researchers with a likelihood of working on topics different from their PhD work. It would also explain why in a number of these publications, CARTA fellows are not first authors, as the publication may not be related to their PhD research.

Further, almost all fellows are doing their PhD while they continue their usual work, albeit some allowance of time-off for their PhD. This may limit some fellows’ ability to publish. There are also different traditions at the CARTA institutions about how PhDs are awarded. Some allow for PhD by publication, and some insist on PhD by monograph and restrict publication from the PhD until the degree is awarded. Again this would explain some of the variations between the outputs of CARTA fellows.

The study indicated a gender dimension in publishing cross-CARTA fellow collaborative papers. The number of male fellows publishing cross-CARTA fellows’ publications was greater than that of female fellows. While gender equity in CARTA has been achieved across the entire programme [16], the early cohorts comprised more men than women. This may explain to some extent, the lower output by women. It is also likely that additional responsibilities traditionally assigned to women such as childcare and other household chores may mean women have less time to work on publications [21,22].

CARTA’s efforts to build cross-cohort, cross-institutional and multidisciplinary collaborations were realised by recruiting fellows from multiple disciplines and different institutions, and organizing cross-institutional and cross-cohort seminars and encouraging cross-institutional registration [16]. We found that this did result in publications between fellows and in particular across institutions and disciplines. While we cannot say this is greater or less than other intervention as we do not have a counterfactual, it does confirm other research which indicated that for collaboration to be effective structured interventions where authors work together to identify common research interests, share ideas, resources, and information are recommended [23,24].

CARTA aims to develop networked research leaders, and earlier studies have described a close relationship between networking and academic career development [25]. Academic networking is described as contributing to cultivating interpersonal relations and identifying common research interests [26,27]. Teamwork contributes to producing an established and networked researcher, which is one of the ultimate aims of CARTA [2,14]. The collaborative publications between CARTA fellows suggest that this skill is being developed in some of the CARTA fellows. The various components of the CARTA program that promote collaboration appear to be related to the research capabilities of the CARTA fellows, a view shared by other authors [1,28].

The cross-institutional publications were encouraging even if limited in number. Importantly, collaborative publications were produced even after fellows had left the formal CARTA program after graduating. This has the potential to sustain cross-institutional collaboration among African partner institutions beyond the CARTA program

Further, the number of cross-cohort publications within and outside fellows’ institutions may be an indication that cross-cohort mentorship was useful, time will tell if this contributes to long-term research collaborations among CARTA fellows. Investing further in this practice can be deliberately encouraged even after the fellowship to enhance peer-learning and research capabilities, and produce quality and productive researchers on the African continent [28,29].

## Limitations

Our analysis was limited to exploring the relationship between CARTA interventions and CARTA fellows’ publication output. As we had no comparison data, we could not draw any inference between CARTA and normal academic activity or other similar programs. However, this does not compromise the observational assessment of the program we are making, and the implications of the findings. Further, we may not have identified every publication of every CARTA fellow since this study relied on CARTA monitoring and evaluation data which is self-reported by fellows.

## Conclusions and recommendations

We conclude that the interventions of the CARTA program have potential to stimulate collaborative-minded researchers on the African continent. We are unable to compare this program to other similar programs, or usual practise when such a program does not exist as we do not have comparative data. Nonetheless, we can conclude that CARTA induced collaborative research publications on health-related issues among African researchers. Of particular interest is that inter-cohort and interdisciplinary publications from within the same institution were a common joint output. This has immediate applicability, as universities can immediately set up mechanisms to facilitate such contacts within their institutions; joint inter-departmental meetings and joint PhD seminars can be initiated at minimum cost as can linking junior PhD students with senior students.

The number of collaborative publications of between fellows, and with non-CARTA researchers suggests that a more concerted effort towards strengthening the intra-African networks may be advised. Investments by CARTA to increase opportunities for its fellows, both doctoral and graduates, to meet and engage in research activities regularly and beyond the fellowship would be beneficial to consolidate further the research networks and increase joint research output among the African institutions. The activities can be in the form of research exchange forums, organized collaborative workshops for joint research and grant proposal development. Avenues could also be created for greater structured engagement of CARTA graduates in activities in other partner institutions. These activities would strengthen the links already established by the fellows during the doctoral fellowships and go beyond the individual fellows to contributing to institutional research collaboration.

These results are significant to the CARTA Program as it moves into its second decade. These results may also inform programs of a similar nature or with similar aims to promote intra-African research collaboration.

## Paper context

Africa needs well trained and networked researchers to respond to its health burden. Collaborative research has proven to respond to that need, as it leads to high and quality scholarly productivity. This study contributes to the knowledge of the process of building a collaborative research mindset among early career researchers in sub-Saharan Africa by investigating the relationship between CARTA program interventions and cross-PhD fellow research output. The study informs programs that aim to promote inter and intra-African research collaboration.
